# Increased Mortality in Schizophrenia Due to Cardiovascular Disease – A Non-Systematic Review of Epidemiology, Possible Causes, and Interventions

**DOI:** 10.3389/fpsyt.2014.00137

**Published:** 2014-09-26

**Authors:** Petter Andreas Ringen, John A. Engh, Astrid B. Birkenaes, Ingrid Dieset, Ole A. Andreassen

**Affiliations:** ^1^NORMENT, KG Jebsen Centre for Psychosis Research, Oslo University Hospital and Institute of Clinical Medicine, University of Oslo, Oslo, Norway; ^2^Division of Mental Health and Addiction, Oslo University Hospital, Oslo, Norway; ^3^Division of Mental Health and Addiction, Vestfold Hospital Trust, Tønsberg, Norway

**Keywords:** schizophrenia, mortality, metabolic syndrome X, physical training, lifestyle, cardiovascular diseases

## Abstract

**Background:** Schizophrenia is among the major causes of disability worldwide and the mortality from cardiovascular disease (CVD) is significantly elevated. There is a growing concern that this health challenge is not fully understood and efficiently addressed.

**Methods:** Non-systematic review using searches in PubMed on relevant topics as well as selection of references based on the authors’ experience from clinical work and research in the field.

**Results:** In most countries, the standardized mortality rate in schizophrenia is about 2.5, leading to a reduction in life expectancy between 15 and 20 years. A major contributor of the increased mortality is due to CVD, with CVD mortality ranging from 40 to 50% in most studies. Important causal factors are related to lifestyle, including poor diet, lack of physical activity, smoking, and substance abuse. Recent findings suggest that there are overlapping pathophysiology and genetics between schizophrenia and CVD-risk factors, further increasing the liability to CVD in schizophrenia. Many pharmacological agents used for treating psychotic disorders have side effects augmenting CVD risk. Although several CVD-risk factors can be effectively prevented and treated, the provision of somatic health services to people with schizophrenia seems inadequate. Further, there is a sparseness of studies investigating the effects of lifestyle interventions in schizophrenia, and there is little knowledge about effective programs targeting physical health in this population.

**Discussion:** The risk for CVD and CVD-related deaths in people with schizophrenia is increased, but the underlying mechanisms are not fully known. Coordinated interventions in different health care settings could probably reduce the risk. There is an urgent need to develop and implement effective programs to increase life expectancy in schizophrenia, and we argue that mental health workers should be more involved in this important task.

## Introduction

Schizophrenia is a major public health concern, causing extensive suffering and a large need for costly treatment and care. Schizophrenia is among the top 10 causes of disability adjusted life years (DALYs) worldwide (1). The standardized mortality rate (SMR) calculated by dividing the observed mortality of a cohort by the expected mortality of an age- and gender-matched cohort of the general population is considerably elevated in schizophrenia. Today, the largest single cause of death in schizophrenia is cardiovascular disease (CVD) (2, 3), similar to the general population. The aim of this paper is to review clinical and epidemiological evidence for increased CVD mortality in schizophrenia, possible causes and mechanisms, available treatments and preventions, and focus the discussion on development and implementation of effective interventions to increase life expectancy in schizophrenia.

## Methods

We conducted a non-systematic review. We used PubMed search terms including “schizophrenia” and “mortality” or “CVD,” “somatic health,” “screening and interventions,” and included publications selected based on knowledge generated from our research in this field as well as clinical experience from working with people with schizophrenia.

## General Mortality in Schizophrenia

Meta-analyses have reported a 2.5- to 3-fold increase in mortality ratios in schizophrenia (4–8), in both inpatient and outpatient settings (9). In a recent survey from Australia including 7000 individuals with psychosis, all deaths in an observation period was registered and SMR was calculated to be as high as 5.5 (65 deaths), with even higher rates for young adults (SMR 15; 8–15 deaths) (10). These mortality ratios are alarming and higher than reported in other similar studies. Possibly, this discrepancy may reflect real variations in SMR in different sub-populations within psychosis spectrum disorders.

Life expectancy for people with schizophrenia is estimated to be about 15–20 years shorter than for the general population (2, 11). A particular cause for concern is that the mortality gap between the general population and schizophrenia seems to have increased during the last decades (8, 12). Death from unnatural causes appears to be 10–20 times higher in schizophrenia than in the general population (2, 4, 13). Suicide and accidents accounted for about 40% of the extra deaths, while 60% were from natural causes (4, 8, 14, 15).

Increased mortality from natural causes is not a recent phenomenon; two large Scandinavian studies from the first part of the twentieth century found significantly increased SMRs of 2.1 (16, 17) and 4.0 (16, 17). The main causes of death were tuberculosis and pneumonia. The high mortality was ascribed to the general disease prevalence and lack of antibiotics but also to the overcrowding, malnutrition, and poor hygiene of large psychiatric institutions.

## Cardiovascular Disease-Related Deaths in Schizophrenia

Several cohort studies addressing the CVD-related mortality have been conducted during the last decade. A large retrospective study from the UK including 46,000 people with a severe mental disorder (schizophrenia-spectrum and -bipolar disorder), and 300,000 controls found the hazard ratios for CVD mortality to be threefold for the age group 18–49 years and twofold for the age group 50–75 years (18). A retrospective register study from Stockholm county in Sweden including 7800 persons with schizophrenia found CVD to be the main cause of death with a SMR of 2.3 and 2.1 for males and females, respectively (13). A more recent prospective national register study from Sweden involving 8300 persons with schizophrenia found hazard ratios for CVD mortality to be 3.3 (12 years reduced life expectancy) for women and 2.2 (15 years reduced life expectancy) for men (19). Another recent prospective study from Sweden, Finland, and Denmark using registers found similar SMRs (range 1.6–2.5) for CVD-related deaths in all the three countries, and life expectancies were 15–20 years shorter than in the general population (11). The increased rates of CVD-related deaths have been confirmed also in autopsy studies (20).

## Possible Causes of Increased Risk for CVD-Related Deaths in Schizophrenia

### Metabolic syndrome and obesity

The metabolic syndrome (MetS) is best seen as a physiological change with a clustering of interrelated metabolic risk factors for developing type-2 diabetes and CVD, as well as a variety of other disorders. The main risk factors are abdominal obesity, elevated blood pressure, dyslipidemia, and insulin resistance, but lately other mechanisms such as endothelial dysfunction, inflammatory activity, and stress are also recognized as important components (21).

Over the last 10 years, schizophrenia has consistently been shown to be associated with a raised prevalence of the MetS (22–25). McEvoy and colleagues (24) reported on baseline findings from 686 persons with chronic schizophrenia in the CATIE Study, where the prevalence of MetS was over 35% in males and over 50% in females. Saari and colleagues (25) found that people with schizophrenia from the Northern Finland 1966 Birth Cohort Study had a fourfold risk of the MetS and that the risk was particularly increased in young individuals. A recent meta-analysis by Mitchell and colleagues investigated the prevalence of specific CVD-risk factors in a schizophrenia population who was either unmedicated (*n* = 1325), in first episode (*n* = 2548), or in the chronic phase (*n* = 24892) to a healthy population. They found that about 25% of the unmedicated and first-episode groups were either overweight, had elevated blood pressure, or dyslipidemia. This prevalence rate is lower compared to the chronic group, but higher compared to an age-matched healthy population (26). However, the same study reported that the prevalence of type-2 diabetes and hyperglycemia was the same in young people with schizophrenia and their healthy peers (26).Taken together, these and other results may suggest that the increased prevalence of type-2 diabetes in schizophrenia is related to treatment with second-generation antipsychotics (AP) (27). However, others have shown that first-episode, drug-naive patients with schizophrenia have impaired fasting glucose tolerance, more insulin resistance, and higher levels of plasma glucose compared to healthy controls (28).

High CVD morbidity is also found in patients with mental disorders other than schizophrenia. In a sample of middle-aged and older patients with psychotic symptoms, the risk for CVD was increased by 60–70% in patients with mood disorder or post-traumatic stress disorder (PTSD) relative to the Framingham 10-year risk of CVD (29). In a Finnish study, the prevalence of MetS in depressed patients was 36% (30). CVD-risk in a sample of people with major depressive disorder or bipolar disorder increased with 50% from pre-treatment to 2-year follow-up (31).

Obesity, part of the MetS, is more prevalent among people with schizophrenia than in the general population (32). Important causes for obesity include excessive levels of food intake, physical inactivity, as well as genetic disposition and an imbalanced interplay between hormones (insulin, leptin, and adiponectin) and several inflammatory markers (including interleukins, the tumor necrosis factor family, and C-reactive protein) (21, 33).

In this review, the different components of the MetS and obesity are further discussed in the specific chapters.

### Lifestyle

#### Tobacco smoking and substance use

Smoking has well-known detrimental effects on CVD-risk profile and mortality (34, 35). Tobacco smoking is about three times more prevalent in people with schizophrenia than in the general population, and the amount of smoking is also higher among smokers (22, 36–38). While tobacco smoking has seen a downturn in the general population (39), smoking rates do not seem to decline in people with schizophrenia (40). In a recent Finnish survey of psychotic disorder, smoking at baseline, along with diabetes, was found to be the most important predictor of 8-year mortality (41). Tobacco-related conditions comprised approximately 53% of total deaths in people with schizophrenia in a recent large cohort study (42).

Prevalence of substance abuse is also high in schizophrenia (38), and this is problematic as substance use in itself is associated with lower psychosocial functioning (43, 44), higher risk of exacerbations, or relapses and may also have direct adverse somatic effects (45–48). Recently, mortality from natural causes was found to be especially high in patients with comorbid substance use disorders (49). However, cannabis use was in one study reported to be associated with decreased mortality in psychotic disorder, which was tentatively associated with anti-inflammatory effects of the endocannabinoid system (50). The most commonly used illicit substance in schizophrenia is cannabis, followed by amphetamines and cocaine (38, 51), although availability and preferences vary across nations and regions.

The increased substance use and smoking in schizophrenia may partly be explained by altered reward-seeking behavior and deficits in the representation of value of stimuli (52, 53). This is in line with the Reward Deficiency Syndrome model proposed for substance abuse (54), which seems to be associated with the pathophysiology of schizophrenia (55) and the biological pathways implicated in nicotine dependence and smoking cessation (56). This model also suggests treatment strategies (54), which may also have implications for interventions targeting other CVD-risk factors.

#### Physical inactivity and poor diet

Schizophrenia is associated with sedentary behavior (57). The engagement in recommended weekly physical activity was estimated to 26% in people with schizophrenia (58), which was lower than in the general population (34%) (59). Other studies applying different assessment tools have also shown that people with schizophrenia are less active (60, 61) and exercise less (62, 63). Low levels of physical activity and lack of physical fitness reduce life expectancy and increase risk for MetS and CVD in the general population (64) as well as in schizophrenia (65). Negative symptoms typical for schizophrenia include lack of initiative and withdrawal from activities, often leading to a physically less active life. Passivity and inadequate motivation might also hinder the pursuit of a healthy diet. Furthermore, the lack of self-insight in one’s own illness (66) might complicate the possibilities for health promoting activities in schizophrenia. During hospital admissions, patients have more symptoms and lower levels of functioning. Activities are often restricted and admissions of longer duration may have considerable impact on activity levels. The nature of an inpatient psychiatric setting has been considered “obesogenic” (67).

Diets rich in calories, fat, and carbohydrates are an important cause for MetS and increased CVD-risk (68). A balanced diet may have potential to reduce the risk for adverse medical conditions (69–71). Unhealthy diets are prevalent in people with schizophrenia (72). Loss of interest in personal hygiene and physical maintenance are also possible reasons. Alterations in the reward system may also contribute to the unhealthy eating patterns (73). There are indications that dietary habits of the mother may affect taste preferences in the offspring (74), and this might add to other factors contributing to unhealthy eating patterns in families.

Poor personal economy is prevalent in schizophrenia (75, 76), and high levels of unemployment is an important reason (77, 78). Negative symptoms and cognitive deficits are the main reasons for work disability (79). Poverty affects the possibilities to engage in a healthy lifestyle, as access to healthy foods and training facilities is expensive.

### Antipsychotic medication

With the introduction of chlorpromazine in 1952, the treatment of schizophrenia was revolutionized, and nowadays medication with AP drugs is considered a central element in the treatment of psychotic disorders (80). However, until second-generation antipsychotics (SGAs) became available in the 1990s, improvement of psychosis often came at the cost of extrapyramidal symptoms and tardive dyskinesia, sometimes causing more subjective suffering to patients than the disorder itself, and making adherence to treatment a great challenge. At the turn of the last century, metabolic disturbances associated with SGA became a major concern in the treatment of severe mental disorders. Several meta-analyses have shown APs to have different liabilities of causing weight gain and other metabolic side effects. On a group level, clozapine and olanzapine are considered to be the most obesogenic, closely followed by quetiapine, with risperidone, sertindole, and typical APs in an intermediate position, while aripiprazole, amisulpride, and ziprasidone seem to carry the lowest risk (81–83).

However, most trials have studied previously treated patients with prolonged illness, often receiving co-medication with drugs with their own obesogenic properties. There are few prospective, long-term studies on the effects of different APs on previously drug-naïve patients on strict monotherapy. Several treatment studies have shown results diverging from the apparently clear-cut panorama of metabolic side effects mentioned above (84, 85). Interestingly, weight gain on olanzapine appeared more rapidly while after 12 months it was the same as haloperidol and risperidone (86). Furthermore, sensitivity to side effects differs with age, gender, and ethnicity, as well as individual factors that are not well understood. In addition, several lines of evidence suggest that SGAs may have exacerbated existing somatic health issues in schizophrenia. Metabolic disturbances, such as diabetes and slow blood sugar uptake, had already been observed and described in the “the mentally insane” of the pre-AP era (87–89). Likewise, the first report on AP-induced obesity, shortly after chlorpromazine had been introduced (90), states that “increase in weight, often considerable, has (previously) been reported with such diverse treatments as insulin shock, cardiazol, and electric convulsive therapy.”

Antipsychotics constitute a heterogeneous group of drugs that may influence appetite control, body composition, and metabolic regulation through several pathways, a variety of underlying mechanisms of action have been proposed. As APs have complex pharmacological actions and interact with most biogenic amine receptors in the brain, a large number of candidate receptors have been targeted. Kroeze et al. (91) showed that the most robust predictor of a drug’s propensity to induce weight gain was its affinity for the H_1_-histamine receptor (H1R). Through this mechanism, clozapine and olanzapine have been shown to cause hyperphagia and weight gain in rats by selective stimulation of the intra-neuronal enzyme adenosine monophosphate (AMP)-activated protein kinase (AMPK) in the hypothalamus, thereby blocking the action of the anorexigenic hormone leptin (92). In accordance with this, findings from one naturalistic study suggest that females with inherent high levels of leptin and high insulin sensitivity might be protected against the obesogenic properties of olanzapine (93). In addition, weight gain and hyperglycemia might be related to SGA effects on inflammatory pathways (94). On the other hand, dyslipidemia has been shown to be significantly associated with olanzapine treatment independently of body mass (95). There are also indications of a direct lipogenic effect of olanzapine caused by increased expression of lipid biosynthesis genes (96).

Despite the obvious risks due to metabolic side effects, AP use has not been consistently shown to increase CVD morbidity or mortality in schizophrenia. Osborn and colleagues (18) concluded that the excess death rates found among mentally ill people could not be explained neither by smoking, social deprivation, nor by the use of AP medication alone, although patients on APs seemed to be at even greater risk than those without. In a recent meta-analysis of drug trials with placebo controls, AP use was not found to be related to increased mortality in schizophrenia, although the higher general mortality in this patient group was replicated (97). Furthermore, two large Finnish registry studies demonstrated *reduced* mortality from all causes in people with schizophrenia on long-term treatment with APs (98, 99). Better somatic care in patients receiving AP medication could be part of the explanation, however, debate continues on the interpretation of these findings and the role of APs in the all-over schizophrenia mortality (41, 100, 101). In addition, sudden cardiac death in schizophrenia has in several studies been associated with the use of APs, most probably related to arrhythmias (102–104). Not every study confirms this picture; Manu and co-workers (105) found that unexpected deaths in people with schizophrenia most likely was due to coronary events and found no hypermortality related to higher levels of AP-treatment.

### Shared pathophysiological mechanisms in schizophrenia and CVD

Several lines of evidence indicate shared underlying pathobiology between schizophrenia and CVD, beyond the effects accounted for by lifestyle and AP medication.

First, this relationship might be due to common genetic factors that contribute to both comorbid medical conditions and mortality in schizophrenia (106). In particular, there seems to be overlapping genes associated with both increased CVD-risk factors and schizophrenia (107, 108). This might explain recent findings that first-episode psychosis patients not treated with AP medication are more likely to have obesity, insulin-resistance, dyslipidemia, and hypertension compared with age-matched healthy controls (26, 109), and the pre-AP era findings mentioned above (see Antipsychotic Medication). Recent molecular studies have indicated that there are abnormalities in the glucose metabolism and insulin signaling pathways in subgroups of unmedicated people with schizophrenia indicating a shared genetic vulnerability between type-2 diabetes and schizophrenia in some cases (110). Second, many neurotransmitters (dopamine, serotonin, and histamine) implicated in schizophrenia also have peripheral effects on pancreatic β-cells and adipocytes, which regulates blood glucose levels, obesity, and lipid levels (111–113). Third, pathways involving the hypothalamic–pituitary–adrenal axis (HPA) might be involved. First-episode psychosis patients have increased cortisol levels (28) possibly due to environmental stress (114) and these alterations in the HPA-axis might account for some of the increased CVD mortality (115). Finally, recent studies have implicated the immune system in severe mental disorders such as schizophrenia (116, 117), as well as in CVD (118), and it is possible that the increased CVD-risk associated with schizophrenia is related to inflammatory mechanisms (119).

### Help seeking and access to somatic health services

Patients with severe mental disorders report greater difficulty and more barriers in accessing primary health care (120). These patient groups also receive poorer quality of somatic health care services from hospitals. Thus, there is an increased risk for serious conditions to be undiagnosed or inadequately treated (121, 122). People with schizophrenia seem to receive less somatic health care also compared to people with other mental disorders (6, 123–125). In schizophrenia, it is shown that treatment and prevention for CVD is suboptimal, and this seems to affect mortality (126, 127). This is supported by an increased mortality due to other somatic conditions besides CVD (15).

In the literature, there are a number of reports of people with schizophrenia who appear to experience little pain despite suffering from painful acute medical conditions, such as myocardial infarction (128) or perforated bowel (129). A meta-analysis of 12 studies on pain perception in schizophrenia indicated reduced pain sensitivity (130). The hypoalgesia was also found in drug-free patients, consistent with clinical observations of reduced sensitivity to pain in schizophrenia reported before the introduction of APs. Pain insensitivity is also found in the healthy relatives of patients with schizophrenia, suggesting a genetic component (131). There is a concern that lack of physical pain sensitivity may result in potentially serious medical conditions passing undiagnosed in schizophrenia (132). There is some evidence that the insula, a cortical structure with extensive connections to different parts of the cortex and the limbic system, is affected in schizophrenia. The insula is important in establishing awareness of the body’s internal state (interoception) and plays a major role in the processes of pain sensitivity (133). Apart from patient-related barriers, inadequately organized somatic care in the mental health services and an existing culture with little focus on somatic health issues in psychiatric or mental health clinics may be an important part of the explanation (134, 135).

## Intervention Studies

### Smoking cessation

Reducing smoking-related mortality will require the delivery of effective anti-smoking strategies. Smoking cessation may have considerable impact on CVD death risk, while just a reduction in smoking does not seem to have the same effect (136, 137). Both pharmacological and psychosocial interventions for smoking cessation have been found to be useful in helping people with schizophrenia quit smoking, but the evidence for a lasting effects is poor (138). Concerns that individuals with severe mental illness might suffer negative mental consequences when quitting smoking seem to have little scientific support (139, 140).

In a Cochrane review of 21 trials (141), pharmacological treatment with bupropion, a dopamine agonist, was found to increase smoking abstinence rates in smokers with schizophrenia. Another pharmacological agent, varenicline, a nicotinic receptor partial agonist, could also facilitate smoking cessation, but with possible psychiatric adverse effects. Psychological interventions such as contingent reinforcement might also help people with schizophrenia to quit or reduce smoking in the short-term. At the present time, there are indications that individuals with schizophrenia can stop smoking with appropriate help, although, convincing evidence that such interventions have a long-term benefit is lacking.

### Increased cardiorespiratory fitness

Patients with schizophrenia have low levels of physical activity (36). Increased physical exercise has beneficial effect on several CVD-risk factors such as body weight and blood lipid concentrations (142) and may reduce mortality (143). Improvement in physical fitness seems possible in schizophrenia (144, 145) and improved physical fitness seems likely to reduce the elevated mortality in this patient group (146). A Cochrane database review concludes that exercise programs are feasible and may improve mental well-being and overall outcome among patients with schizophrenia (145), as well as in mental illness in general (147). Positive results of promoting physical exercise in people with schizophrenia in outpatient and day care settings have also been reported (148–151).

The association between high cardiorespiratory fitness specifically (max oxygen uptake – VO max) and low risk of CVD (independently of weight reduction) is established in healthy men and women (152). Studies have shown that people with schizophrenia have reduced peak oxygen uptake, but are capable of participating in physical exercise programs, which improve their peak oxygen uptake (153, 154). The effect of the improvement seems to be comparable with effects of physical training in healthy controls and patients with CVD (153).

### Direct control of nutrient uptake and expenditure: diet, exercise, medication, and surgery

Obesity is a condition mainly caused by a relative excess intake of calories versus energy expenditure. A focus on these parameters (diet and activity) is therefore a natural starting point for prevention and treatment. Effective weight management may best be reached by the implementation of several measures in an integrated fashion. As there is a complex interplay between the factors responsible for obesity and the other components of the MetS (e.g., hypertension, dyslipidemia, insulin intolerance), effective interventions will be expected to have effects on several CVD-risk factors. Diet modifications may hold some of the same benefits as physical activity for reducing CVD-risk (71). Specific diets (e.g., Mediterranean, nut-enriched) may be favorable in achieving effects on different aspects of the MetS (155–157).

Studies have shown that weight reduction is possible when applying specific intervention programs for individuals with psychotic disorders (149, 158). The combined effect of behavioral intervention, nutritional information, and physical exercise was investigated in a study of people with schizophrenia and body mass index (BMI) exceeding 25. The 3-month intervention program showed significant reduction in weight and BMI, which lasted 12 months (159). Intriguingly, weight reduction was gradual, with increasing weight loss as the program progressed, in contrast to weight reductions patterns in diet programs in healthy individuals, where there is often a larger reduction of weight early on.

People with schizophrenia treated with atypical APs participated in a 1-year multimodal weight control program, comprising nutrition, exercise, and behavioral interventions. Clinically significant reductions in weight and other risk factors for poor somatic health, including hemoglobin A1c were found in the patients who participated in the program. In contrast, patients who did not receive the weight control intervention continued to gain weight (160). A Cochrane review investigating the effects of 23 randomized controlled trials found that modest weight reduction can be obtained in people with schizophrenia with selective pharmacological and psychological interventions both on diet and activity levels (161).

For established obesity or MetS, pharmacological and surgical treatment may be needed and constitute important adjunctive measures. Finding safe and effective pharmacological interventions is difficult. Several drugs have been tested for weight reduction. There is evidence for some efficacy for different classes of drugs currently on the market such as, e.g., lipase inhibitors (Orlistat), antidepressants (Lorcaserin, Bupropion), anticonvulsants (Topiramat), and also Metformin for the treatment of comorbid diabetes (162, 163). However, there are problems with the evidence for sustained effects as most studies have <24 months follow-up. Amphetamine-like appetite suppressants (e.g., phentermin) are now mostly banned in western countries because of the potential for abuse and should probably be avoided in persons vulnerable to psychosis. Sibutramin was withdrawn from the European markets in 2010 because of cardiovascular side effects. There is some evidence that medication and low caloric diet interventions are better than exercise or dietary supplements in maintaining weight loss (164). Statins may be considered for treatment of dyslipidemia in schizophrenia. Available evidence suggests similar treatment response as in the general population (165).

New insights in the metabolism of fats have led to investigation of the role of the hormone leptin in weight loss; however, any possible effect seems to be limited only to individuals with a genetic altered metabolism of leptin (166, 167). Alternative classes of drugs are under investigation for weight reduction in AP-induced weight gain in schizophrenia and may hold promise (168, 169). Studies in schizophrenia are limited and lack data on long-term effects. However, when other efforts to reduce weight gain have failed, the use of medication, and perhaps especially Metformin, seems to have some support in available evidence from systematic reviews and meta-analyses (170–172). However, no medication for obesity has been shown to reduce CVD morbidity or mortality (173).

Bariatric surgery, where parts of the digestive system are removed or bypassed, seems to provide greater weight reduction and higher rates of remission of type-2 diabetes and MetS compared to non-surgical interventions according to a recent meta-analysis limited to 2 years of follow-up (174). There are preliminary indications that people with schizophrenia receiving this kind of surgery may have similar outcomes as the general population (175).

### Clinical monitoring

There seems to be a consensus that in patients receiving AP medications, weight, blood glucose, blood pressure, and lipid levels should be assessed at baseline and monitored regularly. American Diabetes Association et al. (176) recommended that weight should be assessed 4, 8, and 12 weeks after initiating or changing AP medication and quarterly thereafter. Blood pressure, blood glucose, and lipid levels were to be checked 3 months after initiating AP therapy. If normal, blood pressure and blood glucose levels should be assessed every year or more frequently, and lipid levels should be measured every 5 years or more frequently. Several countries have implemented national guidelines with similar recommendations, but the compliance with these guidelines is uncertain (177).

Dealing with the described barriers to adequate somatic health care for people with schizophrenia could include improving availability of personnel skilled in detecting signs of CVD as well as the implementation of routines for checking CVD-risk factors in the mental health care settings. Current evidence makes it relevant to consider implementing such assessments also in people with schizophrenia not receiving AP medication.

If the assessments reveal increased risk factors, several courses of action are possible and should be planned according to the needs of the individual patient. However, important measures will often include change of medication and specific treatment of the present risk factors according to established guidelines for each condition.

## Discussion

The present evidence provides a clear picture of an increased mortality rate in schizophrenia, approximately two to three times higher than in the general population. The majority of the increase is due to CVD. This is a serious health care issue that has to be dealt with. The precise origin of the raised vulnerability to CVD in schizophrenia remains elusive and most likely cannot be attributed to a single mechanism. In the interpretation of the findings of increased mortality, there is a problem that very little research is done on drug-naïve patients, and the full role of AP medications in these associations is thus problematic to assess. At present, most authors agree that the causality is multifactorial. Unfortunately, it is difficult with targeted interventions before the mechanisms are better known. However, the current review of the causative factors indicates that there may be several points of action available for the primary prevention of the increased risk for CVD.

Treatment strategies for reducing the risk-increasing behavior are easy to implement with few side effects, and, thus, potentially widely acceptable. However, even though adequate facilities for physical activities are available, and diet and smoking cessation programs are conducted, patients do not necessarily take advantage of them. Motivation is needed for a change in behavior. Many patients are ambivalent or lack motivation to increase their physical activity. Health care professionals are thus challenged to assist patients in their motivational process. The cognitive technique Motivational Interviewing (178) is increasingly being used in areas of medicine where change in the patients’ behavior is important (179–181), and the results of pilot studies in cardiology are promising (182). The Transtheoretical Model of Change describes the motivational process and seems applicable in physical activity programs in severe mental illness populations (183). There are, however, several other obstacles for lifestyles changes among patients with severe mental illness than the motivational factors. Increased symptom load in general, side effects of medication, socioeconomic problems, staff attitudes, and practical problems related to provision of services also represent barriers (184). These should be included in intervention programs.

Secondary prevention of CVD would focus on detection of the increased somatic risk factors and ease the access to prevention and treatment. Of interest, there is research on possible future psychiatry-specific forms for biological treatment for the increased CVD-risk associated with APs (185). The prescription of medication with potential metabolic side effects should only be done after a careful cost–benefit analysis for each individual. Special attention should be paid for drug-naïve patients as well as patients in older age (80, 186). Psychotropic medication should be followed by a rigorous monitoring of side effects and changes in CVD-risk factors, following established guidelines (187, 188). Findings that the increase in CVD-risk factors occurs early after start of medication should also guide the monitoring (189). As the use of APs is not the only risk factor for increased CVD, AP-free patients should also be monitored for somatic risk factors.

The continuous development and implementation of guidelines is important, but the evidence also points to a demand for somatic proficiency in physicians working in psychiatry and that other mental health professionals contribute. As a group, people with schizophrenia do not receive adequate treatment for CVD, and improving somatic treatment and care in these patients is the first goal. Access to somatic health care should be improved by identifying local health care barriers and thereafter reducing them. Such barriers may exist both in the organization of the services as well as in the minds of politicians, directors, professionals, and patients. The European Psychiatric Association has called for increased cooperation and shared care between the different healthcare professionals to screen and treat CVD-risk factors and diabetes (190). Improved coordination between caretakers and health care professionals is supported by others (190, 191). To obtain an integrated system of care based on a holistic biopsychosocial patient perspective perhaps a complete transformation and reorganization of the current mental health care systems is needed (192). Increasing the awareness of CVD-risk factors among psychiatrists and primary care physicians caring for patients with severe mental illness is also called for (190).

A large fraction of the high mortality in schizophrenia may represent avoidable deaths (193). To meet this serious challenge, better understanding of the underlying mechanisms and improved CVD prevention and treatment are needed. However, better coordination of the health care system is also needed.

## Conflict of Interest Statement

The authors declare that the research was conducted in the absence of any commercial or financial relationships that could be construed as a potential conflict of interest.
